# The Strain at Bone-Implant Interface Determines the Effect of Spinopelvic Reconstruction following Total Sacrectomy: A Strain Gauge Analysis in Various Spinopelvic Constructs

**DOI:** 10.1371/journal.pone.0085298

**Published:** 2014-01-14

**Authors:** Yan Yu, Rui Zhu, Zhi-Li Zeng, Yong-Wei Jia, Zhou-Rui Wu, Yi-Long Ren, Bo Chen, Zu-Quan Ding, Li-Ming Cheng

**Affiliations:** 1 Department of Spine Surgery, Tongji Hospital, Tongji University School of Medicine, Shanghai, China; 2 Julius Wolff Institut, Charité-Universitätsmedizin Berlin, Berlin, Germany; 3 School of Life Science and Technology, Tongji University, Shanghai, China; 4 Institute of Orthopaedics and Traumatology, Shanghai, China; Bascom Palmer Eye Institute, University of Miami School of Medicine, United States of America

## Abstract

**Purpose:**

There is still some controversy regarding the optimal biomechanical concept for spinopelvic stabilization following total sacrectomy for malignancy. Strains at specific anatomical sites at pelvis/sacrum and implants interfaces have been poorly investigated. Herein, we compared and analyzed the strains applied at key points at the bone-implant interface in four different spinopelvic constructs following total sacrectomy; consequently, we defined a balanced architecture for spinopelvic fusion in that situation.

**Methods:**

Six human cadaveric specimens, from second lumbar vertebra to proximal femur, were used to compare the partial strains at specific sites in a total sacrectomy model. Test constructs included: (1) intact pelvis (control), (2) sacral-rod reconstruction (SRR), (3) bilateral fibular flap reconstruction (BFFR), (4) four-rods reconstruction (FRR), and (5) improved compound reconstruction (ICR). Strains were measured by bonded strain gauges onto the surface of three specific sites (pubic rami, arcuate lines, and posterior spinal rods) under a 500 N axial load.

**Results:**

ICR caused lower strains at specific sites and, moreover, on stress distribution and symmetry, compared to the other three constructs. Strains at pubic rami and arcuate lines following BFFR were lower than those following SRR, but higher at the posterior spinal rod construct. The different modes of strain distribution reflected different patient’s parameter-related conditions. FRR model showed the highest strains at all sites because of the lack of an anterior bracing frame.

**Conclusions:**

The findings of this investigation suggest that both anterior bracing frame and the four-rods load dispersion provide significant load sharing. Additionally, these two constructs decrease the peak strains at bone-implant interface, thus determining the theoretical surgical technique to achieve optimal stress dispersion and balance for spinopelvic reconstruction in early postoperative period following total sacrectomy.

## Introduction

There is only sparse literature referring the challenging surgical treatment following sacrectomy for malignant and aggressive lesions. The load transfer between spine and pelvis that follows sacrectomy is highly altered; thus, every surgical reconstruction option should aim to restore spinopelvic continuity to again allow the transfer of loads from the lower extremities through the sacrum toward the spine.

Galveston L-rod technique was used to bridge the spinopelvic gap that results following either sacrectomy or iliosacral disassociation with the use of divergent rods into the pelvis with long lever arms. Either spinopelvic bypass or iliac fixations are the only known techniques to achieve structural continuity and spinopelvic balance. However, the pelvic radiolucencies (halos) around the intrapelvic part of the L-rods that indicated loosening have forced surgeons to replace this L-rods with long iliac screws [1]. Stress concentration at bone–implant interface plays a dominant role to ensure the success of each attempted spinopelvic reconstruction. The overall structural stability following spinopelvic reconstruction was discussed in previous studies [2], [3].

Some authors have analyzed the efficacy of triangular frame reconstruction [4], sacral-rod reconstruction (SRR) [5], and modified Galveston reconstruction [6] following total sacrectomy; they concluded that the risk of instrumentation failure and subsequent implant loosening is less with the SRR method. Various surgical techniques have been developed using the principle of screw-rod fixation (SRF) plus bone graft to achieve solid spinopelvic integrity, although these techniques did not completely eliminate instrumentation failure in the spinopelvic constructs [2].

Quite recently, an improved compound reconstruction (ICR) technique was presented including an anterior bracing frame and posterior four-rods screw fixation [2], and the structural stability of ICR was compared *in vitro* [2] and *in silico* [3] with the three classic techniques: SRR, bilateral fibular flap reconstruction (BFFR) [7], and four-rods reconstruction (FRR) [8], [9]. These previous studies indicated that ICR was the best option to achieve early postoperative stability following total sacrectomy. Subsequently, Mindea *et al.* [10] slightly modified the ICR technique and compared it with the established two-rods construct, resulting in similar trends.

Nevertheless, in the present study local strains at bone-implant interface in four reconstructive methods (ICR, SRR, BFFR, and FRR) were measured because we believe that this feature (local strain) represents an important factor for the success of every surgical intervention. Unlike the numerical studies [4], [5], we believe that biomechanical studies on human cadavers are more persuasive.

The aim of this study is to investigate partial stress distribution and structural balance of different reconstruction methods using strain gauge technique, and determine the optimal technique, considering that lower strains at bone-implant interface safeguards better spinopelvic reconstruction following total sacrectomy.

## Materials and Methods

### Specimen Preparation

**Figure 1:**
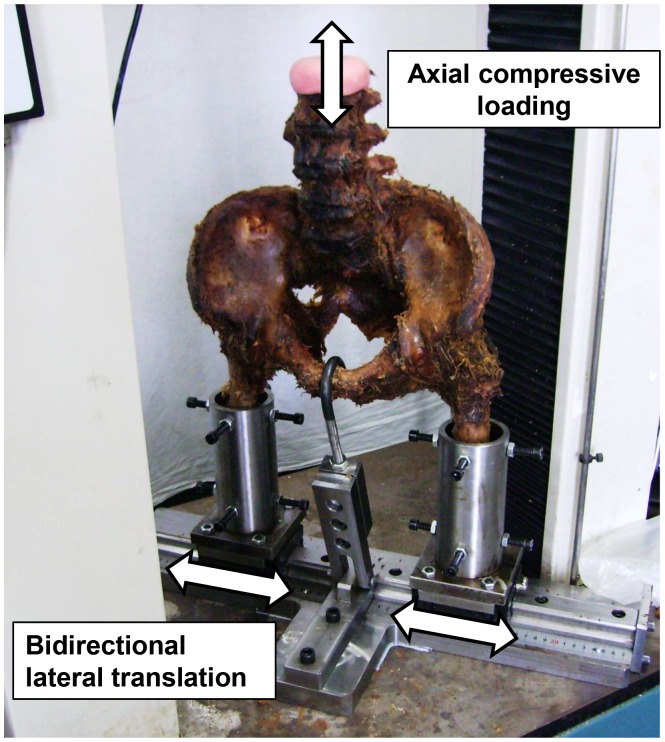
Test equipment. An axial compressive load MTS actuator was placed onto the superior endplate of second lumbar vertebra. Cadaveric femora were firmly fixed into bilateral sleeves of the custom-designed experimental rig. The positioning was maintained by an adjustable metal hook. A preprogrammed universal testing machine controlled the compressive mode.

Six fresh cadaveric specimens (three male and three female, aged 28–76) from second to fifth lumbar vertebrae, pelvis and upper part of femur were obtained, screened for other abnormality (e.g. osteoporosis and bony defect), and meticulously cleaned from excessive soft tissue (e.g., muscles). Care was taken not to damage any of the joint capsules and the supporting ligaments. The upper endplate of the second lumbar vertebra in each specimen was embedded in methyl methacrylate and fixed to the actuator of CSS-44000 MTS machine (CRIMS, Changchun, P. R. China) for the mechanical test. A custom-made rig (No. ZL200720076386.X, CN PAT) [2] allowed bilateral femora for bidirectional translation of the forces in the coronal plane [11] (Fig. 1). To simulate the human physiological posture maintained by muscles in biped stance, an adjustable metal hook was used to firm the triangle plane (which consists of pubic synchondrosis and bilateral anterior superior spine), placed perpendicularly to the horizontal plane through holding the pelvis.

**Figure 2:**
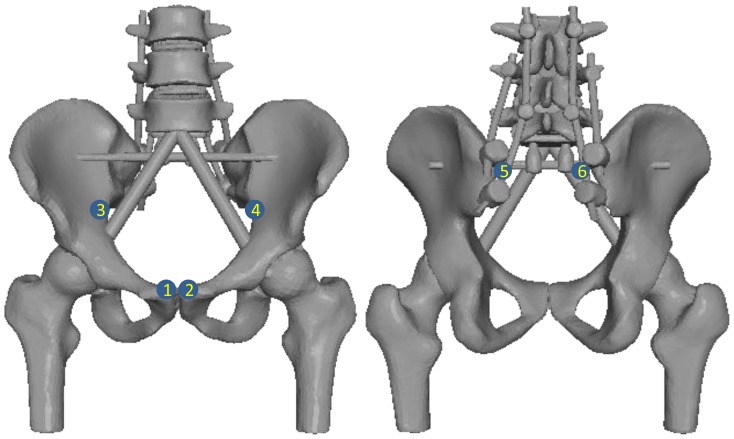
Locations of cemented strain gauges anteriorly (left) and posteriorly (right) in ICR configuration. Points 1 and 2 were bounded onto the bilateral pubic rami. Points 3 and 4 were bounded onto the bilateral arcuate lines. Points 5 and 6 were bounded onto two posterior spinal rods (special note: two medial rods with FRR and ICR configurations).

**Figure 3:**
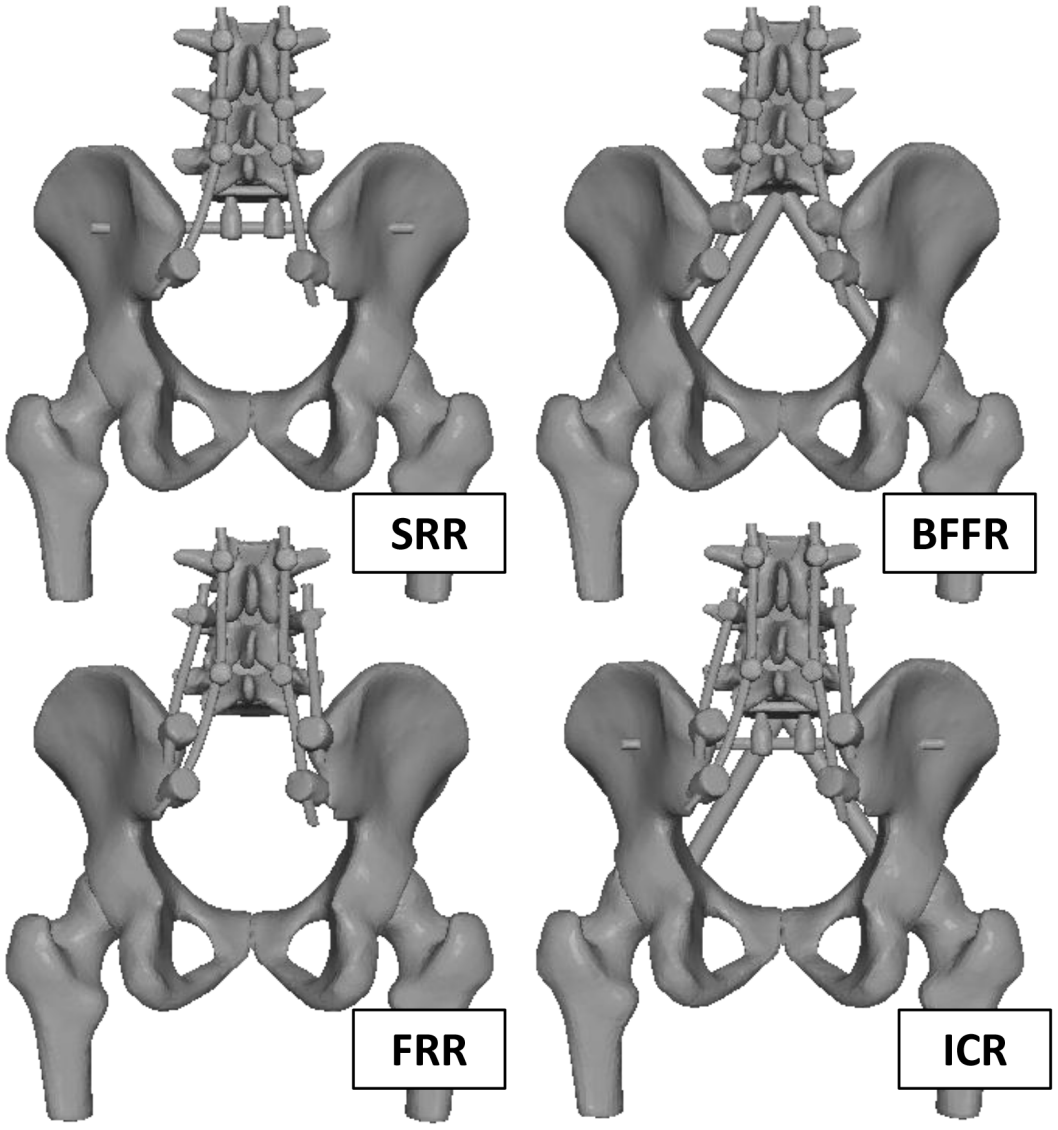
Surgical constructs. Images show four different configurations for SRR, BFFR, FRR, and ICR.

To measure strains at bones and implants, strain gauges (BE120-2AA; ZEMIC, Hanzhong, P. R. China) were cemented to the pelvis at pubic rami bilaterally, arcuate lines and on two posterior spinal rods (Fig. 2). These sites were selected because previous biomechanical reports have disclosed stress concentration at these sites following instrumentation [4], [12], [13]. Micro-strains values were acquired from a static strain tester (DH3818; Donghua Test Technology, Jiangsu, P. R. China). The surgical constructs used for this experiment are shown in Figures 2 and 3.

The same posterior spinal fixation system (Kanghui Medical Innovation, Changzhou, P. R. China) was used for all four constructs but different fixation systems were used for the anterior bracing (Fig. 3). Each specimen was tested under five different configurations in a planned sequence: (1) Intact, or human intact pelvis (non-instrumented); (2) SRR, pelvis instrumented with the sacral rod under the lower endplate of the fifth lumbar vertebra; (3) BFFR, pelvis instrumented with two fibular grafts constructed as a solid triangle between fifth lumbar vertebra and bilateral arcuate lines; (4) FRR, pelvis instrumented posteriorly with four-rods across the lumbo-pelvic junction according to two different pedicle screw trajectories without anterior bracing; and (5) ICR, pelvis instrumented anteriorly with the sacral-rod and the fibular triangular construct, and posteriorly with the alternating screw trajectories technique. In order to minimize the influence associated with the repeated installation and removal of some hardware components, the test sequence above was ordered from lowest to highest destructive step [2]. All constructs were assembled according to manufacturers’ surgical technique manual.

### Non-destructive Biomechanical Testing

For each configuration or construct, the specimen was axially loaded with compression while positioned in double-leg stance. Intact operating conditions were investigated biomechanically to assess human physiological parameters in contrast to standard and controlled programmed compression.

Each specimen was preconditioned with five cycles (0–300 N) at the rate of 2 mm/min before formal testing. An axial compressive ramp load up to 500 N was continuously recorded by MTS actuator transducer, in which 500 N corresponds to erect standing [14]. Stepwise load (five steps of 100 N each) proceeded at a rate of 2 mm/min. Values were obtained by triplicate at every 100 N load and the voltage change data for each gauge were converted to units of micro-strain and averaged over the three repetitions of each test.

### Statistical Analysis

Because the strain response to load was linear (*r* = 1.0), it gave a slope with micro-strain per Newton units that was multiplied by 500 N to give a final strain value. All data, focused to acquire immediate values at 500 N, were analyzed by cluster analysis using SPSS software version 11.5 (SPSS Inc., Chicago, IL, USA). Previous values were presented as mean ± standard deviation. The positive and negative values implied tensile and compressive strain, respectively. After homogeneity of variance and normality tests, the data were analyzed by one-way ANOVA, followed by least significant difference (LSD) analysis for *post-hoc* multiple comparisons. *P*-value <0.05 was considered statistically significant.

## Results

As general prior data, each mechanical testing lasted approximately five hours per cadaveric specimen. During testing, cadavers were sprayed with isotonic water to keep soft tissues moisture.

### Bilateral Pubic Rami Sites

**Figure 4:**
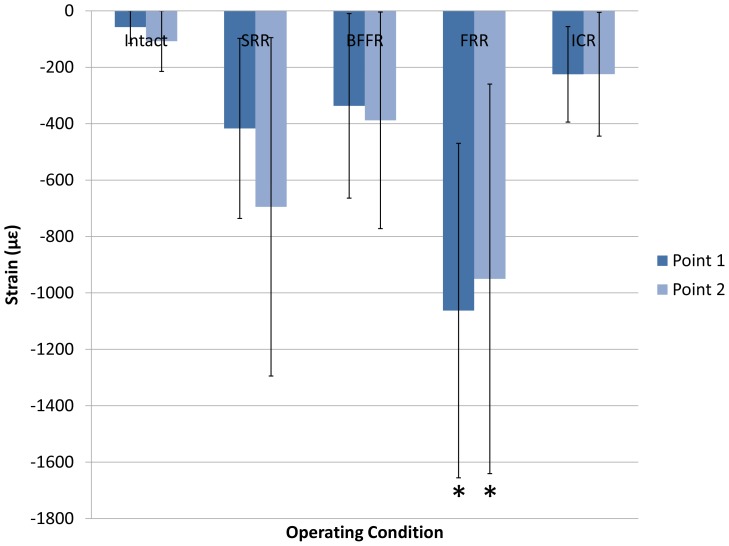
Strains of bilateral pubic rami sites. Histogram shows means and standard deviations of strains at pubic rami bilaterally for the five constructs (intact pelvis, SRR, BFFR, FRR, and ICR). The negative strain values implied compressive strains. The strains under FRR were significantly higher than that under all other conditions (*P<0.05). The strains in ICR presented the best symmetry.

Significant differences among the five configurations were observed at 500 N compressive load (*P* = 0.00 at point 1 and *P* = 0.03 at point 2, Fig. 2). The strain values at pubic rami are shown in Table 1. In this respect, the strains in the ICR showed the best symmetry. The strains under FRR were significantly higher than that under all other constructs (*P*<0.05). No other comparison yielded statistically significant differences (Fig. 4).

**Table 1:** Strains at different sites for different operating conditions.

Operating conditions	Bilateral pubic rami		Bilateral arcuate lines		Two posterior spinal rods	
	Point 1 (µε)	Point 2 (µε)	Point 3 (µε)	Point 4 (µε)	Point 5 (µε)	Point 6 (µε)
Intact pelvis	−57.5±57.4	−107.4±107.2	−238.8±197.4	−218.7±168.0	N/A	N/A
SRR	−416.7±319.2	−694.7±599.9	−140.7±55.6	−148.4±58.5	291.9±252.8	350.6±288.6
BFFR	−336.7±327.2	−388.0±383.9	−82.4±61.0	−131.3±123.8	520.8±516.4	531.1±523.1
FRR	−1062.6±593.0	−950.1±690.6	−146.6±84.5	−159.1±138.0	655.7±651.5	472.8±415.1
ICR	−225.0±169.3	−224.6±219.4	−93.4±56.1	−88.8±60.2	236.0±235.4	191.5±154.8

Note: N/A = Not applicable.

The mean values and standard deviations of static micro-strains (µε) at bilateral pubic rami, bilateral arcuate lines, and two posterior spinal rods sites are shown for five configurations: intact pelvis, SRR, BFFR, FRR, ICR.

### Bilateral Arcuate Lines Sites

**Figure 5:**
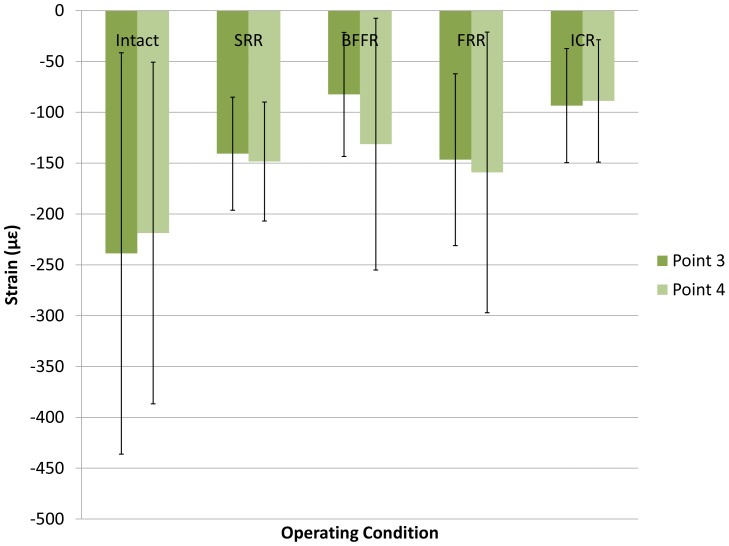
Strains at arcuate lines site bilaterally. Histogram shows means and standard deviations of strains at the arcuate lines for five constructs (intact pelvis, SRR, BFFR, FRR, and ICR). No significant differences were demonstrated. The negative values implied compressive strains. The strains in ICR present the best symmetry.

The strains at bilateral arcuate lines did not differ significantly among all the tested constructs (*P* = 0.13 at point 3 and *P* = 0.52 at point 4) at 500 N (Fig. 5), thus not performing a LSD comparison. All of the values, which indicated a compressive strain in the area of each condition, were negative (Table 1). For its part, ICR was also the most balance configuration.

### Two Posterior Spinal Rods Sites

**Figure 6:**
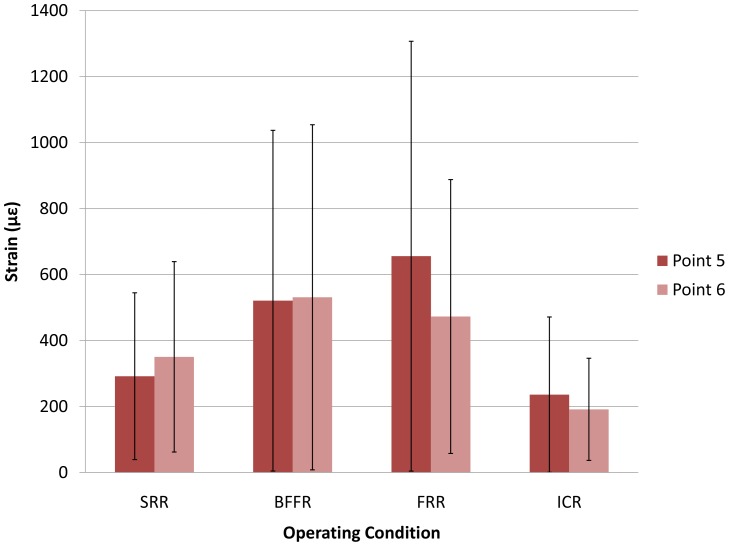
Strains of two posterior spinal rods sites. Histogram shows means and standard deviations of strains at two posterior spinal rods for four constructs (SRR, BFFR, FRR, and ICR). No significant differences were demonstrated. The positive values implied tensile strains. The strains under FRR were significantly higher than that under all other conditions. BFFR produced a little better symmetry than ICR.

The positive values at the posterior spinal rods (500 N) considered as tensile strains, showed no statistically significant differences among the tested constructs (*P* = 0.49 at point 5 and *P* = 0.53 at point 6) (Fig. 6). Therefore, and as explained above, a LSD comparison was not performed. Values are shown in Table 1. From the value bar in Figure 6, BFFR produced a little better symmetry than ICR.

## Discussion

Since the implementation of Galveston L-rod technique, there is still controversy regarding the cause of radiolucent halos around intrapelvic implanted rods [15], [16], rod fatigue fracture [17], and loosening [18]. These complications have some connection to micro-strain variation at bone and instruments from a biomechanical perspective [5]. A variety of techniques has been proposed to avoid these complications [2]–[10], but strain gauge analysis in human cadavers was rarely used.

Previous studies have compared the structural stability of hypothetic reconstructive model with other models [2], [3]. To expand the testing to other parameters of spinopelvic reconstruction, partial strain alternation and structural balance were investigated using strain gauge method. A similar study by Kawahara *et al.* [5], without strain gauge, reported that the risk of instrumentation failure and/or loosening at bone-implant interface was much higher for SRR compared with the intact pelvis.

In accordance to previous similar *in vitro* studies [4], [12], testing sites of high stress concentration in the intact pelvis were selected for locations of strain gauges (bilateral pubic rami, posteriorly to spinal rods). In addition, the arcuate line was also chosen in our study, because it plays an important role in biomechanical architecture of spine-pelvis. Anatomically, the pelvic ring consists of the anterior and the posterior arches in double-leg stance. By these two arches, the load is transferred from the acetabulum to the sacroiliac joint and to the pubic symphysis [19]. Therefore, the arcuate line has become a focal point in stress concentration, which was the same as the pelvic stress nephogram analyzed by 3D finite element method [13]. Finally, two testing sites at both arcuate lines were selected in out testing. According to data of strain gauges retrieved from our study, ICR construct produced better symmetry compared to other three counterparts, but strain values measured at pubic rami were within those recorded both in the intact pelvis and other constructs. Strain magnitudes measured at the arcuate line in all constructs were lower than those in the intact pelvis, whereas in ICR it was the lowest among all four constructs. We speculate that the reason should be the interruption of physiological load bearing arch that follows sacrectomy. The solid bony path provided by sacrum and ilium is replaced by the instrumentation that is composed of spinal rods and iliac screws. Therefore, disperse strains are enhanced at the pubic ramus while they simultaneously decreased at the arcuate line. Merely posterior iliosacral fixation will fail unless the stress is partly divested by the anterior hardware (e.g., sacral rod and fibular graft).

Among four techniques compared in our testing, FRR showed the highest strain not only at the bone interface, but also at the implantation site, while the structural balance in FRR was presented as the least insufficient. Possible explanations for this are: (1) a cross-linking construct underneath the inferior endplate of the fifth lumbar vertebra oriented to support the entire spinopelvic structure was absent; (2) there was no stress-dispersing structure, and thus the load is entirely transferred through the posterior SRF; and (3) although the alternating screw trajectory prevented the pedicle screws removal, the highest stress was generated in the bending of spinal rods. Thereafter, SRR and BFFR presented interestingly complementary phenomena.

Although higher strains at the bone surface were shown following SRR, strains at spinal rods were higher with BFFR, for following reasons: (1) the sacral rod of the SRR effectively prevented lumbar spine down-shifting [2], and thereby vertical stress was diverted from the ilium bilaterally to the lumbar spine through the sacral rod, and the original stress component transmitted to the iliac screw was being reduced [20], [21]; and (2) bilateral fibula graft implantation produced a triangular construct, whose stiffness was better in terms of structural mechanics [2]. Because of the stable and rigid structure associated with BFFR configuration, almost all stresses were diverted to fibular grafts and implants. Nevertheless, BFFR is inferior to SRR in terms of preventing of the lumbar down-shifting immediately postoperation. Because of that, ICR combined the advantages of the other constructs compared in this study. ICR symmetry was better than in the other three constructs and was verified from the left and right strain gauges. The major disadvantage of ICR was its technical difficulty and incidental iatrogenic disturbances.

Usually, patients are permitted to full weight bearing on two crutches 2–3 months postoperation. Based on the results obtained from previous and the current studies [2], [3], immediate postoperative weight bearing would be possible and reasonable with the exception of patients who received FRR.

We do not recommend these four techniques for any lumbo-pelvic reconstruction, but we suggest the individualized approach for carefully selected cases. From this strain gauge analysis, we speculate that patients operated with the SRR technique would suffer from pelvic bone pain possibly due to higher strain concentration at the bone. For its part, BFFR would be associated with a risk of posterior SRF failure and functional exercise should be avoided until complete fusion of the anterior fibula grafts has occurred. Because of the complexity and difficulty of ICR, it should be considered only for patients suffered with serious destruction following complete sacrectomy.

### Study Limitations

Biomechanical studies may have inherent limitations, and especially those performed on cadaveric specimens have the most prominent limitations. Because of the removal of muscle and soft tissues, the stabilization effect provided by ligaments and muscle, that is present in living individuals, was absent in cadavers. With the acknowledgment of inherent limitations, however, cadaver tests can provide useful data if they are run parametrically with a small number of variables being considered. Because human fresh cadavers are difficult to get, the interventional groups were actually small. Due to the hard availability of the samples, only a limited number of samples were used in this study, leading to a limited statistical power. If a greater sample size had been applied, some comparisons lacking statistical difference might be significant. Therefore, the objective of the present study was mainly to compare the trend for four different reconstructions. Additionally, four different reconstructive techniques were performed on the same specimen, the consequential ligaments and bone tissues as well may loosen during repeated trials. As the available number of human fresh cadavers was limited, we did not address this point in the current experimental paradigm. Further biomechanical studies using different specimens will be needed in the future.

To minimize the bone defect caused by repeated screw replacements and the use of the same pedicle for two pedicle screw techniques, the second screw was inserted deeper than the first one (about 1/2-1 thread) with a torque wrench to keep up with the same insertional torque. Due to the good quality of cadaveric bone, a huge torque was still required for removing screws after each testing. Nevertheless, the tissue loosening caused by repeated testing was not solved thoroughly. In addition, the limiting factor of strain gauge circuit sensitivity was found to be the resolution of the analog to digital board which is used. The resolution was such that computed strain values had a resolution of about ±1 µε. Finally, all of the cues *in vivo* may not be accounted in this cadaveric study similar to other *in vitro* biomechanical studies. Therefore appropriate judgment is required when a reconstruction method for clinical cases is selected [2], [3], [10].

Ideally, lumbopelvic structural stability should allow the patient to stand with crutches early during rehabilitation, so the axial compressive load was set at a level of 500 N that corresponds to erect standing [14]. Meanwhile, the alarming load to avoid the specimens’ destruction had been clarified in the pilot study. Due to the limitation of the number of cadavers, one specimen under one configuration was applied to analyze the data of load to failure. Before the pilot study, finite element analysis initially argued that human pelvis following sacrectomy under FRR condition demonstrated the poorest structural stability. Therefore, FRR technique was selected for the set of alarming load (failure with load occurred at 663 N). The empirical results demonstrated that 500 N would be safe for later formal experiment. Taking in consideration the above mentioned limitations, we believe that the experimental data derived from this experimental study may serve in clinical praxis.

## Conclusion

ICR construct produces better symmetry in spinopelvic reconstruction following total sacrectomy and is associated with a low implant failure risk and theoretically less pain. Both anterior bracing frame and the four-rods load dispersion play an important role in load sharing, which decrease the peak strains at bone-implant interface. Therefore, in early postoperative period following total sacrectomy, ICR technique produces optimal stress dispersion and balance for spinopelvic reconstruction.

### Ethics Statement

Ethical approval was obtained from the Human Research Ethics Committee, Tongji Hospital, Tongji University School of Medicine, Shanghai, P. R. China. Before death, all subjects gave written informed consent in native language determining body donation for research reasons.
